# Comparison of Methods to Detect and Measure Glaucomatous Visual Field Progression

**DOI:** 10.1167/tvst.8.5.2

**Published:** 2019-09-11

**Authors:** Alessandro Rabiolo, Esteban Morales, Lilian Mohamed, Vicente Capistrano, Ji Hyun Kim, Abdelmonem Afifi, Fei Yu, Anne L. Coleman, Kouros Nouri-Mahdavi, Joseph Caprioli

**Affiliations:** 1 Stein Eye Institute, David Geffen School of Medicine, University of California Los Angeles, Los Angeles, CA, USA; 2 Department of Ophthalmology, University Vita-Salute, IRCCS San Raffaele, Milan, Italy; 3 Department of Ophthalmology, Cairo University Faculty of Medicine, Cairo, Egypt; 4 Siloam Eye Hospital, Seoul, Korea; 5 Department of Biostatistics, Fielding School of Public Health at UCLA, Los Angeles, CA, USA

**Keywords:** Advanced Glaucoma Intervention Study, Collaborative Initial Glaucoma Treatment Study, glaucoma rate index, guided progression analysis, mean deviation, permutation of pointwise linear regression, perimetry, visual field rate, visual field simulation

## Abstract

**Purpose:**

To compare methods to assess visual field (VF) progression in glaucoma.

**Methods:**

4,950 VFs of 253 primary open angle-glaucoma patients were evaluated for progression with the following methods: clinical evaluation, guided progression analysis (GPA), mean deviation (MD), and visual field index (VFI) rates, Advanced Glaucoma Intervention Study (AGIS) and Collaborative Initial Glaucoma Treatment Study (CIGTS) scores, pointwise linear regression (PLR), permutation of PLR (PoPLR), and glaucoma rate index (GRI). A separate simulated series of longitudinal VFs was assessed with all methods except for GPA and clinical evaluation.

**Results:**

The average (±SD) age of the patients at baseline was 65.4 (±11.5) years. The average (±SD) follow-up was 11.8 (±4.6) years, and the mean (±SD) number of VFs was 16.8 (±7.0). Proportion of series detected as progressing was 65% for PoPLR, 58% for GRI, 41% for GPA, 40% for PLR, 36% for CIGTS, 35% for clinicians, 31% for MD rate, 29% for AGIS, and 22% for VFI rate. Median times to detection of progression were 7.3 years for PoPLR, 7.5 years for GRI, 11 years for clinicians, 14 years for GPA, 16 years for PLR, 17 years for CIGTS, 19 years for AGIS, and more than 20 years for MD and VFI rates. In simulated VF series, GRI had the highest partial area under the receiver operator characteristic curve (0.040) to distinguish between glaucoma progression and aging/cataract decay, followed by VFI rate (0.028), MD rate (0.024), and PoPLR (0.006).

**Conclusions:**

GRI and PoPLR showed the highest proportion of series detected as progressing and shortest times to progression detection. GRI exhibited the best ability to detect progression in the simulated VF series.

**Translational Relevance:**

Knowledge of the properties of every method would allow tailoring application in both clinical and research settings.

## Introduction

Glaucoma is a progressive optic neuropathy characterized by characteristic alterations of the optic nerve head, retinal nerve fiber layer, and visual field (VF). Standard achromatic perimetry is the gold standard for VF assessment, which remains a fundamental test for the diagnosis and care of glaucoma patients. Recognition of longitudinal VF changes allows the timely application of therapeutic measures to preserve visual function and prevent visual disability.^1^ Determination of the rates of progression is important to discriminate fast from slow progressors, because the former group of patients may require more aggressive treatment and more frequent follow-up. Most clinical trials have defined perimetric deterioration as their primary study outcome.^2^–^5^

Detection of true progression and VF rates of change are confounded by intertest variability, often referred to as long-term fluctuation. This fluctuation is higher in glaucoma patients than in healthy subjects.^6^ Age, eccentricity, initial sensitivity, presence of other ocular diseases, and test strategy are other pertinent variables, which affect long-term fluctuation.^7^ A real VF change must exceed the expected amount of noise of the test series and be replicable.^8^ Numerous approaches have been implemented to detect and measure VF deterioration. Subjective judgment is still commonly used in the clinical setting, although its interobserver reliability is unsatisfactory.^9^,^10^ Several statistical models have been proposed to aid the clinician in evaluating perimetric progression objectively, including guided progression analysis (GPA),^11^ rates of change of global indices (i.e., mean deviation [MD], visual field index [VFI], or pattern standard deviation [PSD]),^12^ multivariable regression analysis,^13^ algorithms designed for clinical trials (e.g., the Advanced Glaucoma Intervention Study [AGIS]^2^ and Collaborative Initial Glaucoma Treatment Study [CIGTS]),^5^ pointwise regression analysis (linear or exponential),^14^,^15^ permutation of pointwise linear regression (PoPLR),^16^ and the recently introduced glaucoma rate index (GRI),^17^ Several studies have provided a comparison among some of these methods, but there has been no consensus on which is the single best approach.^7^,^9^,^18^,^19^

We compare here nine published algorithms to detect VF progression, including expert evaluation, GPA, MD and VFI rates, AGIS and CIGTS scores, pointwise linear regression (PLR), PoPLR, and GRI in a cohort of patients and in computer-simulated VF sequences with predetermined rates and patterns of progression.

## Methods

### Study Sample

A total of 4950 VF exams from 253 patients from the Glaucoma Division of the Stein Eye Institute, University of California, Los Angeles (UCLA) were included in this retrospective, longitudinal, observational study. This study adhered to the tenets of the Declaration of Helsinki, was approved by the UCLA Human Research Protection Program, and conformed to the Health Insurance Portability and Accountability Act (HIPAA) policies. Inclusion criteria were as follows: diagnosis of primary open-angle glaucoma with six or more VFs and minimum follow-up of 3 years. All tests were performed with Humphrey Field Analyzer's (HFA) Swedish Interactive Thresholding Algorithm (SITA) Standard 24-2 strategy and size III white stimulus. Reliable exams were defined as those with false-positive rates 15% or less, and false-negative and fixation loss rates 30% or less.^20^ All VF series were assessed for progression with the following methods: qualitative clinical evaluation, GPA, MD rates of change, VFI rates of change the AGIS scoring system, the CIGTS scoring system, PLR, PoPLR, and GRI.

### Simulation Algorithm

Based on models proposed by Spry et al.^21^ and Gardiner and Crabb,^22^ we developed an algorithm in the software environment R (R Foundation for Statistical Computing, Vienna, Austria) to simulate 24-2 VF series. Key steps of the process were as follows:

1. The user specifies the baseline threshold sensitivities, length of follow-up, annual pointwise rates of progression, and number of VF examinations. Tests are equally spaced over the follow-up period, and their frequency is derived as the ratio between the number of VFs and length of simulation;
2. Linear regression analysis is applied at every test location. Independently from the determined rate of progression, an additional decay of 0.1 dB/y is added to simulate age-related decline^23^; and
3. For each location, the noise-free value is replaced by a Monte-Carlo value randomly computed from a Gaussian distribution. The mean of the distribution is equal to the estimated noise-free threshold sensitivity. The standard deviation (SD) is calculated with the function proposed by Gardiner and Crabb^22^:
  \newcommand{\bialpha}{\boldsymbol{\alpha}}\newcommand{\bibeta}{\boldsymbol{\beta}}\newcommand{\bigamma}{\boldsymbol{\gamma}}\newcommand{\bidelta}{\boldsymbol{\delta}}\newcommand{\bivarepsilon}{\boldsymbol{\varepsilon}}\newcommand{\bizeta}{\boldsymbol{\zeta}}\newcommand{\bieta}{\boldsymbol{\eta}}\newcommand{\bitheta}{\boldsymbol{\theta}}\newcommand{\biiota}{\boldsymbol{\iota}}\newcommand{\bikappa}{\boldsymbol{\kappa}}\newcommand{\bilambda}{\boldsymbol{\lambda}}\newcommand{\bimu}{\boldsymbol{\mu}}\newcommand{\binu}{\boldsymbol{\nu}}\newcommand{\bixi}{\boldsymbol{\xi}}\newcommand{\biomicron}{\boldsymbol{\micron}}\newcommand{\bipi}{\boldsymbol{\pi}}\newcommand{\birho}{\boldsymbol{\rho}}\newcommand{\bisigma}{\boldsymbol{\sigma}}\newcommand{\bitau}{\boldsymbol{\tau}}\newcommand{\biupsilon}{\boldsymbol{\upsilon}}\newcommand{\biphi}{\boldsymbol{\phi}}\newcommand{\bichi}{\boldsymbol{\chi}}\newcommand{\bipsi}{\boldsymbol{\psi}}\newcommand{\biomega}{\boldsymbol{\omega}}\begin{equation}\tag{1}\ln \left( {{\rm{SD}}} \right):{\rm{\ }} - 0.081{\rm{\ }} \times {\rm{\ sensitivity\ }}\left( {{\rm{dB}}} \right) + 3.27\end{equation}

To account for higher variability in peripheral test locations, an eccentricity-weighting factor is added to the SD as described by Spry et al.^21^ Specifically,

\newcommand{\bialpha}{\boldsymbol{\alpha}}\newcommand{\bibeta}{\boldsymbol{\beta}}\newcommand{\bigamma}{\boldsymbol{\gamma}}\newcommand{\bidelta}{\boldsymbol{\delta}}\newcommand{\bivarepsilon}{\boldsymbol{\varepsilon}}\newcommand{\bizeta}{\boldsymbol{\zeta}}\newcommand{\bieta}{\boldsymbol{\eta}}\newcommand{\bitheta}{\boldsymbol{\theta}}\newcommand{\biiota}{\boldsymbol{\iota}}\newcommand{\bikappa}{\boldsymbol{\kappa}}\newcommand{\bilambda}{\boldsymbol{\lambda}}\newcommand{\bimu}{\boldsymbol{\mu}}\newcommand{\binu}{\boldsymbol{\nu}}\newcommand{\bixi}{\boldsymbol{\xi}}\newcommand{\biomicron}{\boldsymbol{\micron}}\newcommand{\bipi}{\boldsymbol{\pi}}\newcommand{\birho}{\boldsymbol{\rho}}\newcommand{\bisigma}{\boldsymbol{\sigma}}\newcommand{\bitau}{\boldsymbol{\tau}}\newcommand{\biupsilon}{\boldsymbol{\upsilon}}\newcommand{\biphi}{\boldsymbol{\phi}}\newcommand{\bichi}{\boldsymbol{\chi}}\newcommand{\bipsi}{\boldsymbol{\psi}}\newcommand{\biomega}{\boldsymbol{\omega}}\begin{equation}\tag{2}{\rm{Eccentricity\ weighting\ factor}} = {{\left[ {\sqrt {{{\left( {4.5 - i} \right)}^{2}}} + \sqrt {{{\left( {4.5 - j} \right)}^2}} } \right]} \over {6.5}},\end{equation}

with *i* and *j* being the coordinates of a 10 × 10 grid. According to this model, the variability is proportional to the diminution of threshold sensitivity and the point eccentricity. Values generated below 0 dB or above 36 dB are truncated at 0 or 36 dB, respectively.

The “VisualFields R” package was employed to generate MD, VFI, the total deviation numeric map, and the total deviation probability map for each simulated examination.^24^

### Simulated Settings

**Figure 1:**
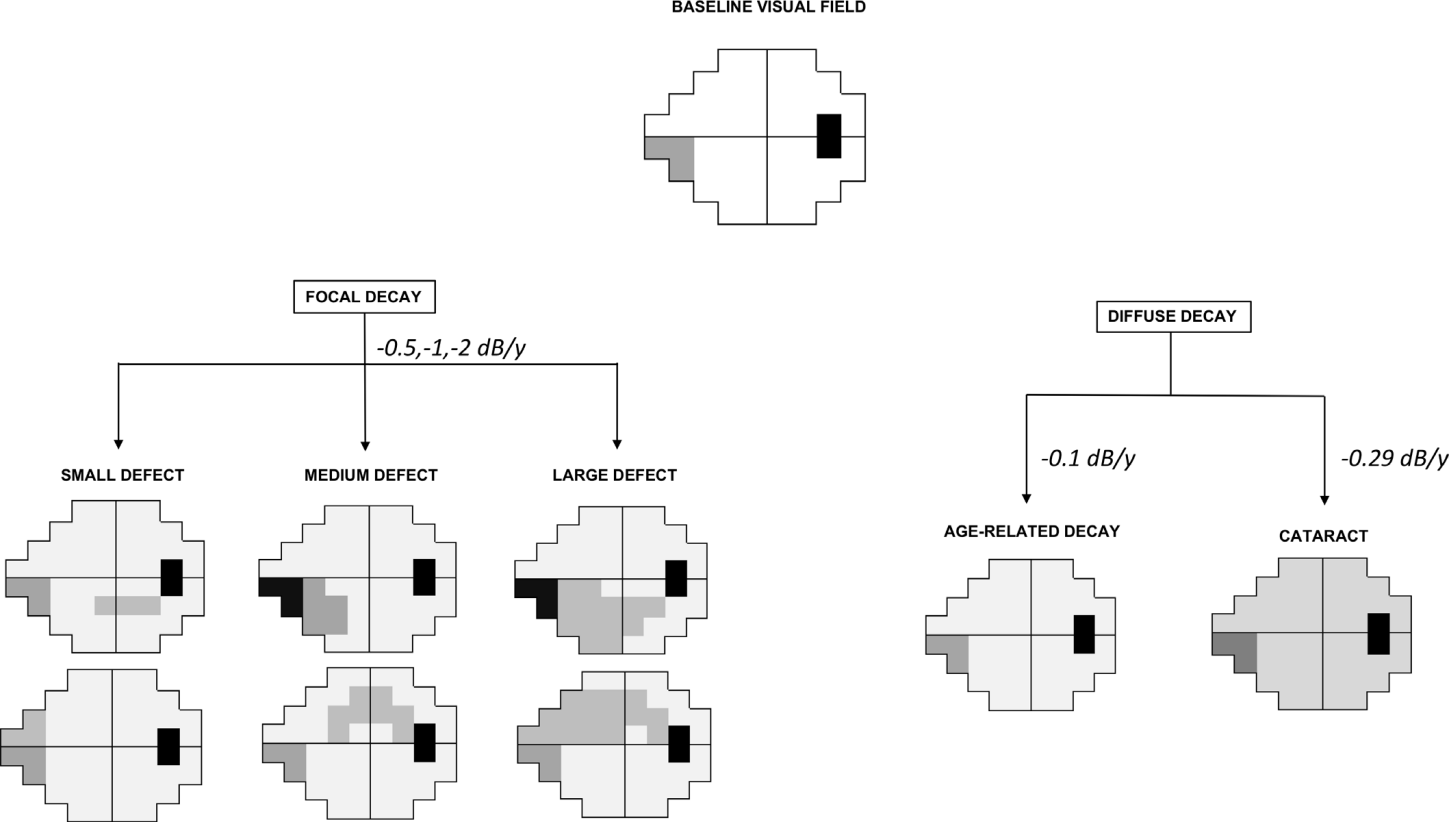
Models generated by computer simulation. Focal or diffuse patterns of progression were applied to two actual baseline VFs with preexisting glaucomatous damage (inferior nasal step and superior arcuate scotoma [not shown]) with different patterns and rates of progressions.

A total of 267,840 VFs belonging to 13,392 simulated eyes were generated, with a ratio of 1:1 between progressing and nonprogressing sequences, in accordance with previous simulation studies.^22^,^25^ Simulation length was established at 9.5 years with a biannual testing frequency for a total of 20 VFs. Baseline age was set at 60 years. Two VF exams from actual glaucoma patients, one with a focal inferior nasal defect and another with a superior arcuate scotoma, were chosen as the two baselines. We then applied two different models of progression, as illustrated in Figure 1:

1. Focal decay, wherein four (small scotoma), eight (medium scotoma), or 16 (large scotoma) locations significantly deteriorate. Simulated defects of small dimensions were represented by a nasal scotoma and a paracentral scotoma. Medium-sized defects were represented by a nasal step and an arcuate scotoma extending to 5° from fixation. Large defects consisted of two broad, inferior and superior, arcuate scotomas. Pattern of focal deterioration were selected by a glaucoma specialist author from VFs of actual glaucoma patients. Three different rates of progression, −0.5, −1, and −2 dB/y, were applied to deteriorating locations in addition to normal age-related decay. The hemifield involved was randomly assigned. Based on the baseline exams, rates, and patterns of progression, 18 scenarios were simulated and each of them was run 372 times, to match the number of eyes in the cohort of patients. All eyes with deterioration of simulated scotomata were considered progressing.
2. Diffuse decay, wherein every location undergoes the same rate of progression. In one model, we applied only age-related decay (0.1 dB/y).^22^ In accordance with previously published results, we specified a rate of progression of 0.29 dB/y to simulate an average rate of decline from worsening cataract.^26^ These two groups of eyes were considered nonprogressing. Based on the two baseline examinations and rate of progression, four scenarios existed for this group, and each was run 1674 times so that the number of progressing and nonprogressing series was equal. The differences between these iterations represent the random noise provided by the model.

### Methods to Detect Perimetric Progression

Nine methods were employed to identify perimetric progression as follows: (1) qualitative clinical evaluation, (2) GPA based on Early Manifest Glaucoma Trial (EMGT) criteria, (3) MD rates of change, (4) VFI rates of change, (5) the AGIS scoring system, (6) the CIGTS scoring system, (7) PLR, (8) PoPLR, and (9) GRI.

All metrics were computed in a sequential fashion, starting at the fifth VF of the series and adding the next examination at every cycle. For the simulated VF examinations, GPA and expert evaluation were not applied. The former is proprietary software and cannot be exactly replicated outside the STATPAC 2 environment (M. Patella, personal communication). The latter is based on SITA printouts, which were not available as output for the simulations. For actual patient data, all modalities were available. Methods to evaluate VFs are explained below, and Table 1 illustrates criteria for VF progression for each method. To assess performance of the methods to identify fast-progressing eyes, we defined eyes with focal damage experiencing a pointwise decay of −2 dB/y as fast-progressors, whereas all other eyes were marked as nonfast-progressors.

**Table 1:** Criteria for Visual Field Progression

Method	Criteria
Expert evaluation	Average score <3
GPA	Threshold deviation outside test–rest variability boundaries from the baseline threshold deviation in ≥3 locations sustained in 3 consecutive VFs
MD rate of change	Significant regression slope (*P* < 0.05) of ≤ −0.5 db/y
VFI rate of change	Significant regression slope (*P* < 0.05) of ≤ −1.8 %/y
AGIS	Score increased of ≥4 points compared to the baseline test, and sustained in at least 3 consecutive examinations
CIGTS	A worsening of ≥3 points compared with the average of the first 2 tests, and sustained in at least 3 consecutive examinations
PLR	Three test locations having a significant regression slope (*P* < 0.01) of ≤ −1 dB/y
PoPLR	Original VF sequence over the 95th percentile compared with the distribution of randomly permutated series
GRI	Score < −6

#### Expert Evaluation

The grading protocol was previously described and is summarized here.^27^ Three experienced clinicians (ALC, JC, KNM) independently assessed 4950 VF examinations from 372 eyes. The evaluation was carried out on the 24-2 HVF single-examination printouts, and graders were masked to all clinical data, results of other scoring systems, and other graders' judgments. Each grader determined progression of each VF series with a semiquantitative scale, with a score of 1 (definite progression), 2 (probable progression), 3 (indeterminate), 4 (probably stable), and 5 (definitely stable). For each eye, an average score from 1 to 5 was calculated. “Progression” and “no progression” were defined as an average score of less than 3 and 3 or more, respectively. Experts also indicated the time when they first judged the VF as progressing, and the average time to progression was determined.

#### Guided Progression Analysis

GPA is proprietary software of the HFA (Carl Zeiss Meditec, Inc., Dublin, CA). GPA is based on similar principles as its earlier version, the Glaucoma Change Probability Analysis.^28^ Briefly, the pattern deviation values on each follow-up VF are compared point-by-point with those of the two baseline exams chosen by the user. If the difference for a given location is significantly higher than test–retest variability at a *P* < 0.05, it is marked with an open triangle. If the worsening of that point is confirmed on two or three consecutive examinations, it is flagged as a half-filled or filled triangle, respectively. VF progression was defined as the deterioration of three or more locations sustained on three or more consecutive examinations, in accordance with the criteria adopted by the EMGT.^3^

#### Mean Deviation and VFI Rates of Change

Rates of change for MD and VFI were calculated with linear regression, and progression was defined as a significant (*P* < 0.05) rate of change of −0.5 dB/y or less and −1.8%/y or less, respectively. The value of 1.8% for VFI was chosen because it corresponds approximately to the 0.5 dB/y value used for MD.^29^

#### The Advanced Glaucoma Interventional Study (AGIS) Scoring System

The AGIS scoring system has been previously described.^2^ VF grading is based on number, depth, and spatial distribution of depressed locations with a final score that ranges from 0 (normal) to 20 (end-stage disease). Progression was defined as a score that increased by four or more points compared with the baseline test, sustained in at least three consecutive VF examinations.

#### The Collaborative Initial Glaucoma Treatment Study (CIGTS) Scoring System

This system takes into account each significantly depressed location and the two most significant neighboring points on the total deviation probability map.^5^ Like the AGIS, the CIGTS score ranges from 0 (normal) to 20 (end-stage disease). A worsening of three or more points compared with the average of the first two tests and sustained for three consecutive examinations was defined as progression.

#### Pointwise Linear Regression

Ordinary least squares of the raw threshold sensitivities over time was performed for each of the 52 VF pointwise series. The slope of the regression line, expressed in decibels per year, was defined as the pointwise linear rate of change. The presence of three pointwise series having a significant regression slope (*P* < 0.01) of −1 dB/y or less was defined as progression.

#### Permutation Analyses of Pointwise Linear Regression

The PoPLR algorithm has been published by O'Leary and colleagues.^16^ For each patient's VF sequence, PLR was performed on the total deviation data. Data belonging to every location were combined to generate a global score, called S_obs_, with a truncated product method, which is a generalization of the Fisher method and allows to combine *P* values derived from each pointwise series.^30^ The patient's original VF sequence was then randomly reordered up to 5000 times, and a global score, called S_p_, was obtained from each permutated series. Finally, S_obs_ was compared with the S_p_ distribution, and the statistical significance was derived from the ranking of S_obs_ within the S_p_ distribution. A *P* value of less than 0.05 was labeled as progression.

#### Glaucoma Rate Index

The GRI algorithm has been extensively described elsewhere.^17^ Briefly, each pointwise sequence was classified as decaying or improving depending on its a priori linear trend. Based on this categorization, pointwise exponential regression (PER) was computed for each series. For locations with a negative trend, the following formula was applied: *y = e*^(*a+bx*)^, where y is the threshold sensitivity (dB), a is the constant, b is the slope (regression coefficient), and x is the time (years). For locations with a positive trend, the following formula was used: *Y−y = e*^(*a+bx*)^, where Y is the normal age-matched threshold sensitivity + 2SD, y is the threshold sensitivity (dB), a is the constant, b is the slope, x is the time (years). Outliers were removed with the sequential application of Cook's distance and the Studentized residual tests. After carrying out PER, two values were obtained, pointwise rate of change (PRC) and the 90% confidence interval (CI) of the slopes. The former indicates the rate of change of each pointwise sequence, expressed as the percentage of the entire perimetric range corrected for age and location. A GRI score is generated by summing the PRC values from locations with significant negative (decaying) and positive (improving) rates across the VF series in an individual eye. The summed value is then normalized from a maximum rate of decay (−100) to a maximum rate of improvement (+100). A GRI value of less than −6 was defined as progression.^17^

### Statistical Analysis

The proportion of series determined as progressing was defined as the percentage of progressing eyes defined by each algorithm. Eyes meeting progression criteria according to a given method at any follow-up visit were defined as progressing. Because there is no gold standard to define perimetric progression in a cohort of glaucomatous patients, the proportion of series detected as progressing is used as a surrogate measure of sensitivity. Summary results were expressed as a mean ± SD, unless specified otherwise. As an alternative to a very complicated Venn diagram, the UpSet technique was used to graphically demonstrate progression detected by each method alone and their intersections.^31^ Time to the first detection of progression was assessed with Kaplan-Meier curves, differences across methods were compared with Cox's regression shared frailty model in R software, and multiple comparisons were adjusted with the Benjamini-Hochberg test.^32^

#### Evaluation of Agreement Among Methods

Pairwise agreements among the methods were measured with Cohen's kappa statistic. Values of k between 0.00 and 0.20 indicate slight agreement, fair agreement between 0.21 and 0.40, moderate agreement between 0.41 and 0.60, substantial agreement between 0.61 and 0.80, and almost perfect agreement 0.81 or more.^33^

#### Evaluation of Internal Consistency

Internal consistency was evaluated by counting the number of flips from one state to the other (i.e., progressing and not progressing) for each method, as the VF series proceeded over time. For each method, the flips analysis was carried out only on those eyes progressing according to that method, and “stable” eyes were excluded to prevent the influence of the detection rate of each technique on the consistency estimate. The number of flips for the various methods was compared with the Friedman test, and pairwise comparisons were performed with Dunn's multiple comparisons as a post hoc test.^34^,^35^

#### Prediction Ability

We calculated the agreement with Cohen's kappa statistic between status at the last visit with the entire and the half-time VF sequences for each technique as a measure of prediction ability. Because data on progression were available starting at visit five, those eyes with nine VFs or less (*n* = 32) were excluded from this particular analysis.

#### Simulation Data

Receiver operator characteristic (ROC) curves were generated for the trend-based methods with the known simulated model as the reference. ROC curves were calculated with the continuous values of the trend-based methods at the last visit of the simulated series. Two different ROCs curves were made. Both curves had simulated focal decay as the progressing reference eyes. One ROC curve defined nonprogressing as the age-related decay group only, while the second ROC curve defined nonprogressing as the cataract decay group in addition to the age-related decay group. Partial areas under the curves (AUROCs) were calculated to determine the average sensitivity between 90% and 100% specificity. The sensitivity and 1-specificity values for AGIS, CIGTS, and PLR at the last visit were calculated and plotted as points on the plot.

The sensitivity and specificity in simulated series with the progression criteria used in the clinical cohort of patients were also calculated for trend-based methods. Computer-simulated series, which met the clinical criteria for progression at any simulated time point, were labeled as progressing, while the remaining ones were considered stable.

For comparison of detection of fast progressing eyes, ROC curves were generated with simulated eyes having a focal decay of −2 dB/y as the progressing group and all the other simulated sequences as nonprogressing. Partial AUROCs were calculated to determine the average sensitivity between 90% and 100% specificities. The sensitivity and 1-specificity values of AGIS, CIGTS, and PLR at the last visit were calculated and plotted as points on the plot.

The partial AUROCs values were reported in the uncorrected form. Partial AUROCs were compared in pairs with a stratified bootstrap test,^36^ and multiple comparisons were adjusted with the Benjamini-Hochberg test.^32^

All statistical analyses were performed with R software and GraphPad Prism software 6.0 (GraphPad Software, Inc., San Diego, CA). The R package “pROC” was used to build the ROC curves on the simulated data and to compare partial AUROCs.^36^

## Results

### Patient Cohort

**Figure 2:**
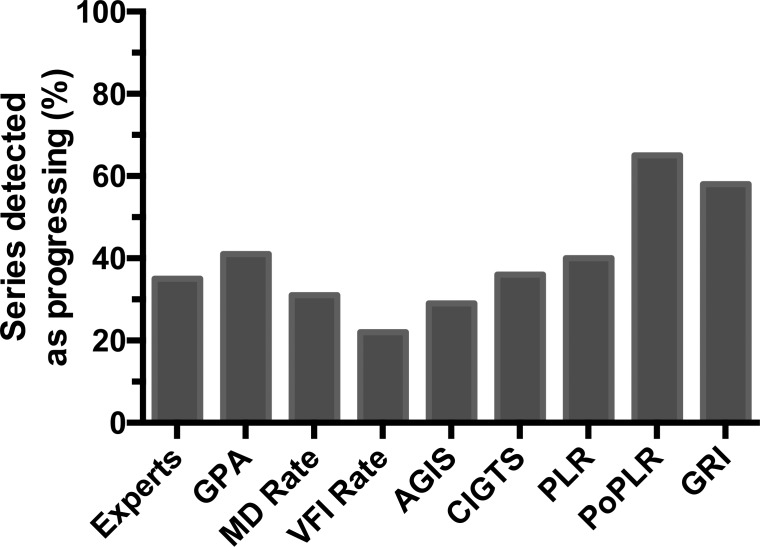
Proportion of series detected as progressing of each method calculated from actual patient data.

Table 2 reports demographic and clinical baseline data for the participants. The average (±SD) age of the patients at baseline was 65.4 (±11.5) years, and the average (±SD) follow-up time was 11.8 (±4.6) years. The mean (±SD) number of VFs was 16.8 (±7.0), and the mean (±SD) frequency of testing was 1.5 (±0.3) VFs/y. As shown in Figure 2 and Table 3, PoPLR and GRI showed the highest proportion of series detected as progressing, followed by GPA, PLR, CIGTS, and expert evaluation, which had moderate proportion of series detected as progressing. MD rate, AGIS, and VFI rate had considerably lower proportions of series detected as progressing.

**Table 2:** Demographic and Baseline Clinical Data of the Study Participants

Variable	Patients
*N* of patients/eyes	253/372
Eye, right/left	186/186
Baseline age, years, mean ± SD	65.4 ± 11.5
Initial MD, dB, mean ± SD	−5.4 ± 5.4
Final MD, dB, mean ± SD	−7.9 ± 7.0
*N* VFs, median (IQR)	15 (11–21)
Follow-up, y, median (IQR)	11.0 (7.5–15.9)

IQR, interquartile range; MD, mean deviation; SD, standard deviation; VF, visual field.

**Table 3:** Measures of Diagnostic Properties of the Different Methods

	Experts	GPA	MD Rate	VFI Rate	AGIS	CIGTS	PLR	PoPLR	GRI
Patients									
Progressing	131	151	114	83	109	135	148	242	217
Nonprogressing	241	221	258	289	263	237	224	130	155
Detection rate % (95%CI)	35.3 (30.3–40.1)	40.6 (35.6–45.6)	30.7 (25.9–35.4)	22.3 (18.1–26.6)	29.3 (24.7–34.0)	36.3 (31.4–41.2)	39.8 (34.8–44.8)	65.1 (60.2–69.9)	58.3 (53.3–63.4)

Eyes meeting progression criteria according to a given method at any follow-up visit were defined as progressing.

**Figure 3:**
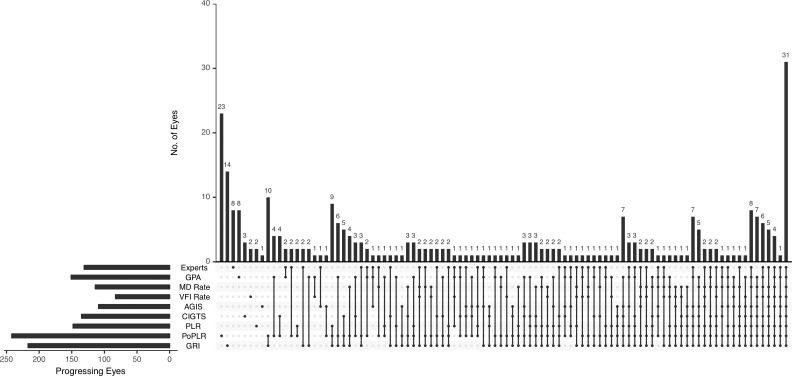
UpSet plots of eyes judged as progressing according to each method in the patient cohort. The horizontal histogram (bottom left) illustrates the total number of progressing eyes for each method. The matrix (bottom) shows selected methods as black dots. When an intersection between two or more methods is displayed, those methods are marked as black dots and connected by a solid, black line. The vertical histogram represents the number of eyes experiencing progression according to a single method, or the intersections between two or more methods. In 75 eyes, none of the methods detected progression.

Figure 3 presents the detection of progression according to each method and their mutual intersections in the patient cohort. Notably, eyes progressing according to all the methods represented the most numerous group (31 eyes). In 75 eyes, none of the methods detected progression.

**Figure 4:**
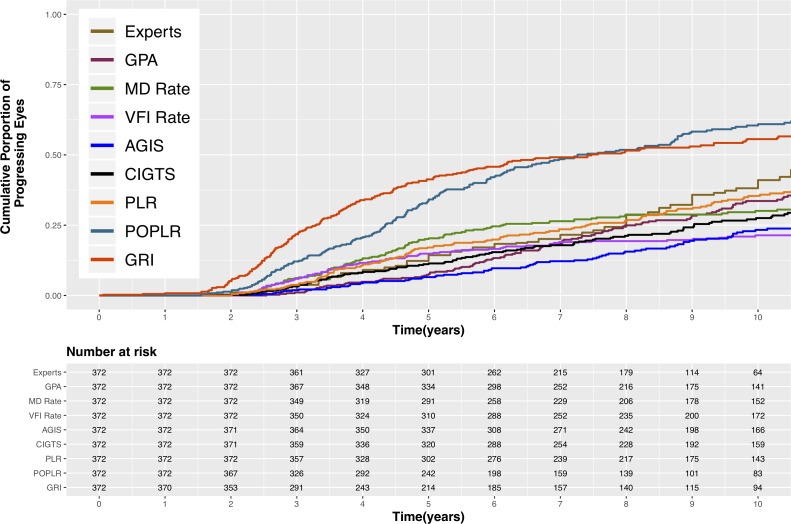
The cumulative proportion of progressing eyes according to each method in the patient cohort.

The cumulative proportion of progressing eyes according to each technique is illustrated in Figure 4. In the patient cohort, the median time to detection of progression was similar for PoPLR (7.3 years) and GRI (7.5 years), and was considerably longer for the other methods: 11 years for clinical experts, 14 years for GPA, 16 years for PLR, 17 years for CIGTS, and 19 years for AGIS. MD rate and VFI rate median times were more than 20 years. Because less than 50% of patients showed progression with MD rate and VFI rate in the study timeframe, the median time could not be calculated. Time to progression was significantly shorter for PoPLR and GRI than for every other method (*P* < 0.001), whereas the difference between these two methods was not significant (*P* = 0.97). VFI rate and AGIS scores required significantly longer times to detect progression compared with all other methods (*P* < 0.01), and did not significantly differ from each other (*P* = 0.12). In addition, PLR detected progression significantly faster than MD rate (*P* = 0.04) and CIGTS (*p* = 0.04). None of the other pairwise comparisons were statistically significant.

Pairwise agreement between methods for the patient cohort is given in Table 4. MD and VFI rates were the only methods displaying substantial pairwise agreement. GRI exhibited moderate agreement with PoPLR (k = 0.575), PLR (k = 0.558), CIGTS (k = 0.424), and MD rate (k = 0.460), while exhibiting a fair agreement with other methods.

**Table 4:** κ Values of the Different Methods to Identify Visual Field Progression in the Cohort of Patients^a^

a Darker shades indicate higher κ values.

The prediction ability of each method, defined as the agreement between results based on the half-time follow-up versus the entire follow-up, is shown in Table 5. Trend-based methods based on global indices (VFI rate and MD rate) showed the highest, albeit moderate, agreement; all other methods exhibited fair agreement.

**Table 5:** Concordance Between Progression Detected at the Last Visit With the Entire and First Half of the Visual Field Sequences in the Cohort of Patients

	Experts	GPA	MD Rate	VFI Rate	AGIS	CIGTS	PLR	PoPLR	GRI
Entire sequence, *n* (%)									
Progressing	126 (37.1)	107 (31.5)	55 (16.2)	50 (14.7)	84 (24.7)	92 (27.1)	98 (28.8)	189 (55.6)	111 (32.7)
Nonprogressing	214 (62.9)	233 (68.5)	285 (83.8)	290 (85.3)	256 (75.3)	248 (72.9)	242 (71.2)	151 (44.4)	229 (67.7 )
Half sequence, *n* (%)									
Progressing	n/a	41 (12.1)	45 (13.2)	32 (9.4)	32 (9.4)	36 (10.6)	39 (11.5)	103 (30.3)	79 (23.2)
Nonprogressing	n/a	299 (87.9)	295 (86.8)	308 (90.6)	308 (90.6)	304 (89.4)	301 (88.5)	237 (69.7)	261 (76.8)
Cohen's κ	n/a	0.33	0.46	0.50	0.38	0.34	0.36	0.35	0.35

n/a, not applicable. Thirty-two eyes were excluded from this analysis because VF series was shorter than 9 VFs.

**Figure 5:**
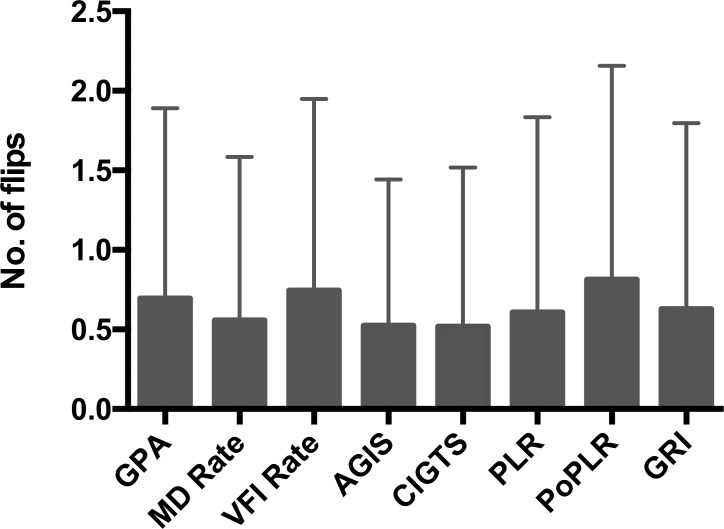
Internal consistency evaluated with number of flips (i.e., changes from progressing to stable and vice versa during the follow-up period) for each method in the patient cohort. Differences among the various methods were nonsignificant (P = 0.17). Bars and error bars indicate the means and standard deviations, respectively.

Internal consistency (Fig. 5), as evaluated by the number of flips over the course of follow-up between progressing and nonprogressing status, was similar among the methods (*P* = 0.17).

### Computer-Simulated VF Sequences

**Figure 6:**
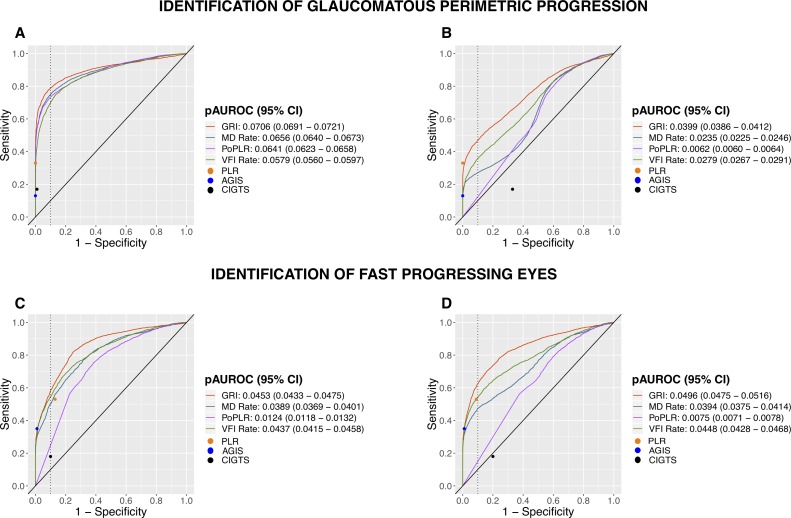
ROC curves relative to the known simulated model for detection of glaucomatous perimetric progression excluding (A) and including (B) eyes with simulated cataract, and for the discrimination of fast progressors excluding (C) and including (D) eyes with simulated cataract. Vertical dotted line indicates a value of 1-specificity of 0.1. Sensitivity/(1-Specificity) values of AGIS, CIGTS, and PLR at the last simulated examination are shown as points on the plot.

All trend-based methods exhibited high diagnostic precision to discriminate between eyes with focal glaucomatous VF loss and with age-related decay (Fig. 6). GRI showed the best partial AUROC (0.0706), followed by MD rate (0.0656), PoPLR (0.0641), and VFI rate (0.0579). Each curve differed significantly from all the others (*P* < 0.0001), except for the pairwise comparison between MD rate and PoPLR (*P* = 0.09). The introduction of eyes with simulated cataract (Fig. 6) considerably hampered the discriminatory abilities of all the trend-based methods, with GRI having the highest partial AUROC (0.0399), followed by VFI rate (0.0279), MD rate (0.0235), and lowest by PoPLR (0.0062).

Table 6 illustrates sensitivity and specificity for the various methods with the progression criteria applied in the cohort of patients. With these criteria, the sensitivity values in simulated data resembled the proportion of series detected as progressing in the cohort of patients, with PoPLR having the highest value, followed by GRI, PLR, CIGTS score, VFI rate, MD rate, and, far beyond, AGIS score. In terms of specificity evaluated on simulated series, AGIS, VFI rate, MD rate, PLR exhibited high values even in the presence of simulated cataract (≥88%). The inclusion of cataract-related decline in the pool of simulated eyes caused a marked specificity drop for the CIGTS score (97% to 66%). PoPLR also had a considerably lower specificity compared with all other methods and performed much worse in the presence of simulated cataract decay. GRI had intermediate specificity values, and was much less affected by cataract simulated decay than PoPLR.

**Table 6:** Measures of Diagnostic Properties of the Different Methods for the Computer Simulated VF Series With the Same Progression Cutoff Used in the Cohort of Patients

	Experts	GPA	MD Rate	VFI Rate	AGIS	CIGTS	PLR	PoPLR	GRI
Without “cataract”									
Sensitivity % (95%CI)	n/a	n/a	25 (24–26)	27 (26–28)	18 (17–19)	29 (28–30)	54 (52–55)	92 (91–93)	76 (75–77)
Specificity % (95%CI)	n/a	n/a	96 (95–97)	94 (93–95)	100 (100–100)	97 (96–97)	96 (95–97)	47 (45–49)	58 (56–60)
With “cataract”									
Sensitivity % (95%CI)	n/a	n/a	25 (24–26)	27 (26–28)	18 (17–19)	29 (28–30)	54 (52–55)	92 (91–93)	76 (75–77)
Specificity % (95%CI)	n/a	n/a	90 (90–91)	90 (89–90)	99 (99 –99)	66 (65–67)	88 (87–89)	23 (22–24)	45 (44–46)

Simulating series meeting progression criteria according to a given method at any point were defined as progressing.

The ROC curves to distinguish fast-progressing from nonfast-progressing eyes showed a similar pattern both without and with the cataract group (Fig. 6). Specifically, GRI had the best performance, followed by VFI rate, MD rate, and, lowest by PoPLR. Interestingly, the introduction of the cataract group marginally affected the partial AUROCs of all the methods, except for PoPLR, which declined more significantly from 0.012 to 0.008. Each curve differed significantly from all the others (*P* < 0.05).

## Discussion

We compared nine published methods to detect glaucomatous perimetric progression: qualitative clinical evaluation, GPA, MD rate, VFI rate, AGIS and CIGTS scoring systems, PLR, PoPLR, and a recently introduced index, GRI. We evaluated the proportion of series detected as progressing, time to progression detection, pairwise agreement of methods, prediction ability, and internal consistency in a cohort of primary open-angle glaucoma patients. Moreover, computer-simulated VFs with predetermined rates and patterns of progression were used as an external reference to test diagnostic performance and to estimate sensitivities and specificities of the tested methods. Agreement among the methods ranged from fair to moderate, except for the MD versus VFI rates, which expectedly displayed substantial concordance. GRI and PoPLR had the highest proportion of series detected as progressing and were the algorithms that detected perimetric progression the earliest. AGIS score, MD rate, and VFI rate had the lowest proportion of series detected as progressing. All methods showed good and similar internal consistency. We investigated the performance of the various trend-based approaches (i.e., GRI, PoPLR, MD, and VFI rates) to detect perimetric progression. All these methods discriminated “glaucomatous” focal decay from the age-related decline well, and the differences across the partial AUROCs were small. When we added a cataract-simulated decline in the nonprogressing reference group, the diagnostic properties of all the methods considerably diminished, with MD rate and, especially PoPLR, most severely affected. We also explored the performance of various approaches for identifying fast progressors in computer-simulated VFs. GRI had the best partial AUROC, followed by VFI rate, MD rate, and PoPLR. Inclusion of the cataract group in this setting had a negative impact only on PoPLR.

In short, GRI and PoPLR had the highest proportion of series detected as progressing, and detected glaucomatous progression the earliest in the cohort of patients, but the latter performed poorly in the computer simulations, especially with the introduction of simulated cataract decay. AGIS, MD rate, and VFI rate showed the lowest proportion of series detected as progressing and were the slowest to detect progression.

Although numerous approaches to detection of glaucoma deterioration have been proposed, timely determination of perimetric progression remains a challenging task for practitioners. None of the available techniques can be considered a gold standard. Subjective evaluation of VF series by the clinician is the most widely employed method, especially in the clinical setting, because it is fast, inexpensive, and independent of a digital environment.^9^ However, its intrinsic subjectivity and lack of standardization are drawbacks. Several studies revealed a disappointing level of agreement among experienced observers, despite good intraobserver reproducibility.^10^,^37^ Also, subjective evaluation is likely driven by the fast component of VF decay.^27^

Event-based analyses identify single events of statistically significant change relative to baseline tests. The proprietary GPA of the HFA is a commonly used event-based analysis and was used by EMGT for detection of glaucomatous progression. It is able to identify worsening test locations with as few as three tests after the two baseline exams. Its ability to detect progression depends on the magnitude of pointwise test–retest variability, which is known to be high at damaged test locations. GPA does not provide a robust estimate of pointwise VF rates of decay.^38^

The AGIS and CIGTS scoring methods are two event-based analyses, which have been employed in large, randomized, controlled trials.^2^,^5^ Heijl and colleagues^39^ compared the properties of EMGT, AGIS, and CIGTS scoring systems with expert evaluation as a reference method. They found that EMGT criteria were more sensitive and identified progression faster than AGIS and CIGTS, albeit less specifically. AGIS and CIGTS criteria had similar diagnostic properties when compared with each other. Vesti et al.^18^ came to the same conclusions with computer-simulated VFs as a reference. Nouri-Mahdavi et al.^19^ compared the performance of an earlier version of GPA, called Glaucoma Change Probability Analysis (GCPA), the AGIS score, and PLR to predict VF progression, and found that GCPA detected true clinical progression slightly more often than the other two methods, with false-positive prediction rates between 1% and 3%.

Our results, both in the patient cohort and in the computer-simulated data, corroborate these previous findings. In our study, AGIS had one of the lowest proportion of series detected as progressing and required a long time to detect progression; conversely, it displayed high specificity and internal consistency. These results are not surprising, because the AGIS method applies stringent criteria, and a considerable, sustained amount of change is required to trigger progression. Mayama et al.^40^ found that an increase of AGIS score of four or more points maintained in two rather than three consecutive tests raised the sensitivity to approximately 50% with negligible change in the specificity. Among the three event-based algorithms in this study, GPA had the highest proportion of series detected as progressing with similar prediction ability and consistency. Because GPA runs on proprietary software, it is not available in the simulated environment, and we were not able to estimate its specificity in relation to the other methods. Previous studies reported an overall high specificity for GPA, although patients with higher test–retest variability and unreliable examinations can experience higher percentages of false-positive alerts.^41^,^42^ The performance of CIGTS was intermediate between AGIS and GPA. The specificity of the CIGTS score was severely affected by the introduction of the cataract-related decay in the computer simulation. This finding is not surprising; indeed, the CIGTS score is based on the total deviation probability map, which is highly influenced by media opacity.^43^ Both AGIS and CIGTS scoring systems were developed for patients with more advanced glaucoma than the ones included in our study, and this may explain their low detection rates in this setting.

Trend-based linear regression models are widely accepted as valuable tools for serial VF analysis because they are simple to calculate, can provide a global rate of change, and have a reasonably good ability to predict future outcomes.^38^ MD and VFI are well-known global VF indices and their linear rates are easily measured; VFI rates of change are provided by HFA's GPA software.^38^ However, MD is relatively insensitive to progressive glaucomatous VF loss and has poor specificity in clinical environments.^44^,^45^ The MD rate of change quantifies overall VF loss, so localized but potentially clinically important changes may be missed entirely or confounded by generalized media effects, such as worsening cataract or cataract surgery, which are common events in glaucoma patients.^44^ Another global index, VFI, fares no better, and becomes unreliable in advanced stages of glaucoma.^46^ Gardiner et al.^45^ reported that as the duration of follow-up and number of VFs increase, it becomes difficult to rely solely on linear models. This is because progression often occurs in nonlinear patterns, especially as the disease severity and its treatment change. Although VFI provides predictive capability with extrapolation, it assumes a linear rate of worsening, and is affected by the same drawbacks as MD.^46^ Global indices have the potential to provide an estimate of VF decay at the expense of loss of spatial information that may be important to clinical decision-making. In the current study, both MD and VFI rates were characterized by low proportion of series detected as progressing and were the slowest to detect progression, although they had better prediction ability than any other method. VFI rate exhibited even lower proportion of series detected as progressing in the cohort of patients, and it performed worse than MD rate in the simulated environment. Gardiner and Demirel^12^ compared the performances of three global indices (i.e., MD, VFI, PSD), and found similar results to ours, with MD rate detecting progression sooner and more frequently than the VFI rate. When we included the cataract group in our computer simulations, however, an opposite scenario occurred with VFI rate performing considerably better than MD rate. This finding is not unexpected because VFI would be expected to be less influenced by cataract and cataract surgery.^47^

Among the trend-based methods, those performing a regression of individual pointwise series over time have potential advantages over those based on global indices. By treating each test location individually, they allow the identification of small focal glaucomatous changes, which may be otherwise missed; as a consequence, pointwise methods have higher sensitivity (but lower specificity) than MD or VFI rates of change.^7^ Another positive aspect of pointwise trend-based methods is the preservation of the spatial information, which provide insight in the patterns, in addition to the rates, of progression.^7^ PLR is undoubtedly the most commonly used pointwise trend-based method and the first to be commercially available in the Progressor software (OBF Labs UK Ltd, Wiltshire, UK). In our study, PLR had a performance similar to GPA in the cohort of patients, and exhibited intermediate sensitivity, but high specificity, in the simulated series. PoPLR is an evolution of PLR that may outperform simple PLR.^16^ PoPLR is independent of data format, and allows for comparison of different instruments, follow-up protocols, and test strategies.^48^,^49^ Our results indicate that PoPLR has a high proportion of series detected as progressing and detects progression early. In the computer simulations, PoPLR was the poorest performing trend-based method, especially when simulated cataract was included in the analysis. These drawbacks are likely related to the assumptions made by the algorithm. PoPLR is generated by comparing a series of VFs of a patient with a maximum of 5000 randomly permutated sequences, assuming that a nonprogressing eye should not differ from the null distribution. However, healthy eyes experience physiological age-related decline and decay from cataract development, and this could be detected as significant progression when compared with the randomly permutated sequences by the algorithm. In an attempt to limit such phenomenon, PoPLR is calculated from the total deviation values, which indicate the difference between the patient's VF and a normal reference VF based on the patient's age. Age-corrected normal thresholds, however, have high interindividual variation, especially in the midperipheral and peripheral locations and follow a non-Gaussian distribution.^23^ O'Leary et al.^16^ reported that PoPLR had a low percentage of false-positives, but these results were obtained with randomly permutated sequences as true nonprogressing sequences, not taking into account the aforementioned factors. In a prospective study by Redmond and colleagues,^48^ PoPLR labeled as falsely progressing almost one-third of healthy subjects followed over a mean time of 5 years; when PoPLR was calculated from the pattern deviation values (rather than the total deviation ones), the false-positive rate was null, reinforcing the idea that this method is severely impaired by the diffuse, paraphysiological VF decay caused by aging and cataract development. Although calculating PoPLR on the pattern deviation values may seem a potential solution to increase its specificity, it is well known that pattern deviation values tend to underestimate VF progression, and may be misleading in the case of very early glaucoma because of a ceiling effect, as well as in severe glaucoma where it can underestimate diffuse generalized damage.^29^,^41^

We recently described a novel trend-based model to assess glaucoma progression, called GRI.^17^ In contrast with PLR and its derivatives, GRI is based on a pointwise exponential regression, in accordance with previous findings that the exponential model fits perimetric measurements and predicts future changes better than linear models.^14^,^50^ GRI has potential advantages, including discriminating ability for fast-progressors, assessment of improvement, and an intuitively interpretable display. In our cohort of patients, GRI had high proportion of series detected as progressing and detected progression faster than other methods and as frequently as PoPLR. On the computer simulation, GRI had the highest partial AUROC both for detection of perimetric progression and discrimination of fast progression compared with the other trend-based methods.

Because GRI and PoPLR exhibited high rates of progression detection, the cutoffs used in this study correspond to points of the ROC curve with high sensitivity and low specificity as shown in Table 6. It should be noted that considerations regarding specificity differ in the population setting versus the clinical setting. The former considers a large group of individuals, and the latter a single patient. In the clinical setting of an individual patient, an astute examiner will bring to bear all the data collected by a complete historic and physical examination to increase the specificity of the determination. On the other hand, more restrictive cutoffs could be employed in those scenarios that require higher degrees of specificity, such as in the case of interventional clinical studies.

Chauhan et al.^51^ defined fast progression and catastrophic progression as a MD worsening rate between 1 and 2 dB/y and more than 2 dB/y, respectively, and they found these conditions in 4.3% and 1.5% in patients under clinical care, respectively. In their study, they identified fast-progressors with MD rate rather than VFI rate, because ceiling effects may potentially hamper the latter's loss of sensitivity because it relies on pattern deviation, and there is discontinuity in case of severe VF damage.^29^,^51^ In our study, VFI rate performed better than MD rate to identify fast-progressors. Neither approach, however, retains spatial information and may be quite insensitive to fast, but focal, deterioration.^52^ In the current study, GRI displayed the best capability to identify fast progressing eyes. PoPLR, which also retains spatial discrimination, performed considerably worse in this regard.

Several studies revealed a fair to moderate level of agreement among methods, including some of those employed in the present study.^19^,^53^,^54^ Our data are consistent with the previous findings. Only 8% of the eyes were judged unanimously as progressing, whereas 20% were deemed as stable by all the methods. In a recent study, Saeedi and colleagues^55^ evaluated the agreement among six methods (PLR, PoPLR, MD, and VFI rates, CIGTS, and AGIS scoring systems) to detect glaucomatous VF progression on a large cohort of patients, and found that eyes labeled as progressing and stable by all the methods were 2.5% and 41.5% of all series, respectively. These results are largely different from ours, and may be explained by various factors, such the shorter follow-up length and more stringent progression criteria for some of the trend-based methods used by Saeedi et al.^55^ Furthermore, the authors have chosen questionable cutoffs for some of the trend-based methods.^56^ For MD rate, Saeedi and colleagues^55^ used a cutoff of −1 dB/y, so that eyes having a rate of progression faster or slower than this threshold value were categorized as progressing or stable, respectively. However, this is quite a high cutoff value, previously used to distinguish between fast progressing and slower progressing eyes, rather than between progressing and stable eyes.^51^ Because MD and VFI are highly correlated, a corresponding cutoff for VFI rate would have been −5.4%/y, which is considerably higher than the one used by Saeedi and colleagues (−1%/y).^29^ It is evident that progression detection strongly depends on the method employed (and cutoff used for each individual method). In our study, MD rate and VFI rate were then only pair to show substantial agreement; this finding is not surprising because the two indices are highly correlated, and we chose equivalent decay rates to define progression.^29^ GRI and PoPLR, the two most sensitive methods in the current study, revealed one of the highest agreement, as exemplified by their conspicuous intersection in the UpSet graph (Fig. 3). On the other hand, the two most specific methods (AGIS and VFI rate) did not exhibit such agreement. Once again, this is not unexpected because several studies have shown discrepancies between event- and trend-based analyses, suggesting that they identify distinct aspects of perimetric change.^11^,^57^ Medeiros and colleagues^11^ proposed a Bayesian hierarchical model to combine event- and trend-based approaches, and they reported that the combined approach outperformed each method used alone. The combination of more than one method may represent a viable option to integrate complementary information from individual algorithms, possibly mitigating their drawbacks.

The present study has limitations. Its retrospective nature dictated that not all eyes had VFs performed at the same frequency, and this can affect the time to detect progression.^58^ Many methods to detect glaucoma progression have been published, and the relationship between untested methods remains undetermined. Nevertheless, we evaluated a considerable number of methods with different strategies (i.e., subjective evaluation, event-based analysis, trend-based analyses), established methods (i.e., AGIS, CIGTS, GPA, MD rate, VFI rate, PLR), as well as novel and promising ones (i.e., PoPLR, GRI). In the simulations, some indices (i.e., MD, VFI, AGIS, CIGTS) were based on calculations carried out with the normative database of the ‘VisualFields' package, and they might differ slightly from the values generated by HVF's software.^24^ Computer simulation is a strategy to obtain an external gold standard, but may oversimplify real and more complex disease progression. Additionally, only a few of all the myriad of possible baseline examinations, patterns, and rates of progression are included in these simulations. GPA and expert evaluation were tested only in the cohort of patients, and were not available in the simulated environment.

In conclusion, we provide a comprehensive comparison of different approaches to assess perimetric glaucoma progression. PoPLR and GRI had the highest proportion of series detected as progressing, and detected glaucomatous progression the earliest; AGIS, MD rate, and VFI rate exhibited the lowest proportion of series detected as progressing and were the slowest to detect progression. Of all the methods tested, GRI had the best ability to detect glaucomatous progression and fast-progression in the simulated VF sequences. Knowledge of the properties, advantages, and limitations of every method would allow tailoring application in both clinical and research settings.
